# Procalcitonin-guided antibiotic treatment in patients with cancer: a patient-level meta-analysis from randomized controlled trials

**DOI:** 10.1186/s12885-024-13160-2

**Published:** 2024-11-28

**Authors:** Claudia Gregoriano, Yannick Wirz, Ashley Heinsalo, Djilali Annane, Konrad Reinhart, Lila Bouadma, Mirjam Christ-Crain, Kristina B. Kristoffersen, Pierre Damas, Vandack Nobre, Carolina F. Oliveira, Yahya Shehabi, Daiana Stolz, Alessia Verduri, Beat Mueller, Philipp Schuetz

**Affiliations:** 1 Medical University Department, Kantonsspital Aarau, Aarau, Switzerland; 2 Department of Clinical Research (DKF), Faculty of Medicine, University of Basel, Basel, Switzerland; 3 IHU PROMETHEUS, Raymond Poincaré Hospital (APHP), INSERM, Université Paris Saclay Campus Versailles, Paris, France; 4 Department of Anesthesiology and Intensive Care Medicine, Jena University Hospital, Jena, Germany; 5 Médecine intensive-réanimation, AP-HP, Hôpital Bichat-Claude Bernard, Paris, France; 6 Division of Endocrinology, Diabetology and Clinical Nutrition, University Hospital Basel, Basel, Switzerland; 7 Department of Oncology, Aarhus University Hospital, Aarhus, Denmark; 8 Department of Intensive Care, University Hospital Liège, Liège, Belgium; 9 Department of Internal Medicine, Medical School and University Hospital, Universidade Federal de Minas Gerais, Belo Horizonte, Brazil; 10 Department of Intensive Care, Monash Medical Centre, Melbourne, VIC Australia; 11 Clinic of Respiratory Medicine, Faculty of Medicine, University of Freiburg, Freiburg, Germany; 12 Respiratory Unit, Department of Surgical and Medical Sciences, University of Modena and Reggio Emilia, Modena, Italy; 13 University Department of Medicine, Kantonsspital Aarau Tellstrasse, Aarau, CH-5001 Switzerland

**Keywords:** Procalcitonin, Cancer, Antibiotic treatment, Meta-analysis

## Abstract

**Background:**

Use of serum procalcitonin (PCT), an inflammatory biomarker for bacterial infections, has shown promising results for early stopping antibiotic treatment among patients with respiratory infections and sepsis. There is need for additional data regarding effectiveness and safety of this concept among patients with cancer.

**Methods:**

Individual data of patients with a documented diagnosis of cancer and proven or suspected respiratory infection and/or sepsis were extracted from previous trials where adult patients were randomized to receive antibiotic treatment based on a PCT protocol or usual care (control group). The primary efficacy and safety endpoints were antibiotic exposure and 28-day all-cause mortality.

**Results:**

This individual-patient data meta-analysis included 777 patients with a diagnosis of cancer from 15 randomized-controlled trials. Regarding efficacy, there was a 18% reduction in antibiotic exposure in patients randomized to PCT-guided care compared to usual care ([days] 8.2 ± 6.6 vs. 9.8 ± 7.3; adjusted difference, − 1.77 [95% CI, − 2.74 to − 0.80]; *p* < 0.001). Regarding safety, there were 72 deaths in 379 patients in the PCT-guided group (19.0%) compared to 91 deaths in 398 participants in the usual care group (22.9%) resulting in an adjusted OR of 0.78 (95% CI, 0.60 to 1.02). A subgroup analysis showed a significant reduction in mortality in patients younger than 70 years (adjusted OR, 0.58 [95% CI, 0.40 to 0.86]).

**Conclusion:**

Result of this individual patient meta-analysis from 15 previous trials suggests that among patients with cancer and suspected or proven respiratory infection or sepsis, use of PCT to guide antibiotic treatment decisions results in reduced antibiotic exposure with a possible reduction in mortality, particularly among younger patients.

**Supplementary Information:**

The online version contains supplementary material available at 10.1186/s12885-024-13160-2.

## Introduction

Among patients with bacterial infections, early initiation of antibiotic therapy reduces mortality and morbidity and is thus a cornerstone of patient management [1]. Nevertheless, prolonged exposure to antibiotics is associated with the risk of antimicrobial resistance [2], and other side effects, which in turn increases morbidity and mortality [3, 4]. In this context, the use of the blood biomarker procalcitonin (PCT) has gained attention as a means to individualize treatment to the need of a patients, particularly among patients with respiratory infection [3] and sepsis [4]. PCT increases during bacterial infections and decreases once the infection is controlled by the immune system [5, 6]. PCT kinetics are thus helpful to early stop antibiotics if a patient shows clinical recovery and a drop in PCT levels by at least 80% from its peak [7]. Use of a PCT protocol has shown to reduce antibiotic exposure without negatively effecting clinical outcome in patients with different types of infections, particularly respiratory infections and sepsis [3, 7–10]. In addition, several analyses also suggested that use of PCT to guide antibiotic treatment also improves microbial resistance [11] and healthcare costs [5, 12]. Importantly, some trials and meta-analyses also found that use of PCT to guide antibiotic treatment resulted in lower mortality and improved patient outcomes [2, 13–16].

Still, the concept of using PCT to guide antibiotic treatment has yet to be validated in some specific patient populations, including patients with cancer - a population that is especially susceptible to complications arising from both the underuse and overuse of antibiotics. In patients with cancer, optimal use of antibiotics is challenging due to the immune-compromising effects of different anti-cancer treatments and immune-activating effects of many cancers with an increase in cytokines and inflammation markers [17–19]. For patients with cancer, multiple studies have found that PCT serves as a more reliable indicator of infection than other biochemical markers, including C-reactive protein (CRP) and white blood cell count (WBC) [20–22]. Nevertheless, elevated levels of PCT not related to infection have been observed in certain cancer types, including medullary C-cell carcinoma and lung carcinoma [23–25], as well as in patients undergoing cancer surgery [26, 27]. It is important to note that these studies were primarily observational and constrained by the absence of a definitive gold standard for identifying infections that require antibiotic treatment. To address this limitation, randomized controlled trials (RCT) are essential to determine whether the incorporation of PCT can enhance the antibiotic management of patients with cancer. Herein, we conducted an individual-patient data meta-analysis focusing on patients with cancer from previous randomized trials to investigate the safety and efficacy of using PCT to guide antibiotic treatment decisions compared to usual care.

## Methods

### Patient population and trial selection

Trial selection and data collection were performed following a protocol published in the Cochrane Library [28] and the report was prepared according to PRISMA individual participant data (IPD) guidelines [29, 30]. We used individual data from patients with documented cancer and proven or suspected infection admitted to the intensive care unit (ICU), emergency department (ED)/medical ward and primary care included in previous trials where patients were randomized to a PCT-guided antibiotic treatment approach (PCT-group) or usual care (control group). We excluded all patients without a documented cancer diagnosis. Detailed definitions are presented in the Table S1.

For the analysis, we used the updated individual patient database from (2017) from their inception date until 2022. The initial protocol published in the Cochrane Library [14] delivered the basis of study selection and data collection. The report was arranged by following the Preferred Reporting Items for Systematic Review and Meta-Analysis and Meta-Analysis individual participant data guidelines [29, 30]. Updates concerning the trial search were undertaken in 2022 in cooperation with the Cochrane collaboration and took place in all databases from their initiation date. We searched a number of databases for trials including the Cochrane Central Register for Controlled Trials (CENTRAL; Feb 10, 2017, Issue 1.), Embase (1980 to 2022) and Medline Ovid (1966 to 2022). There were no exclusion of records based on language restrictions. Furthermore, all references in the trials were assessed for eligibility by two independent authors in accordance with titles, abstracts and full text reports. Risk of bias was assessed in regard to allocation concealment blinded, outcome assessment, follow-up for mortality, adherence to PCT algorithm in PCT group follow up time, consistent with the Cochrane methodology. These included selection bias, performance bias, detection bias, attrition bias and reporting bias. In the need for further information, it was directly acquired from the investigators. Detailed information about risk of bias assessment has been published beforehand [14] and are presented in Table S2.

### Endpoints

The main efficacy and safety endpoints were defined as antibiotic therapy exposure (in days) and 28-day all-cause mortality. Additional secondary endpoints included length of hospital (LOS) and ICU stay within 28 days post randomization. In trials not reporting outcomes up to 28 days, we used the time until hospital discharge. In this case, censoring was used for surviving patients with a follow-up < 28days for the time- to-event analyses.

### Statistical analysis

For the main efficacy and safety endpoints, coefficients and odds ratios (ORs) and 95% confidence intervals (CIs) were calculated using multivariable hierarchical linear and logistic regression [31, 32]. Variables in the multivariate analysis included treatment arm, age, gender, and type of infection. To control for within- and between-trial variability, a “trial” variable was added to the model as a random effect. Analyses followed the intention-to-treat principle by analysing patients in groups to which they were randomized.

Pre-specified subgroup analyses were conducted for treatment setting (ICU, ED/medical ward, primary care), type of infection and level of organ dysfunction (Sequential Organ Failure Assessment (SOFA). We tested for subgroup effects by adding interaction terms to the statistical model. All statistical analyses were performed using Stata version 17.0 (College Station, Texas, USA) and Review Manager version 5.3.

## Results

### Results of systematic search and characteristics of included trials

**Fig. 1:**
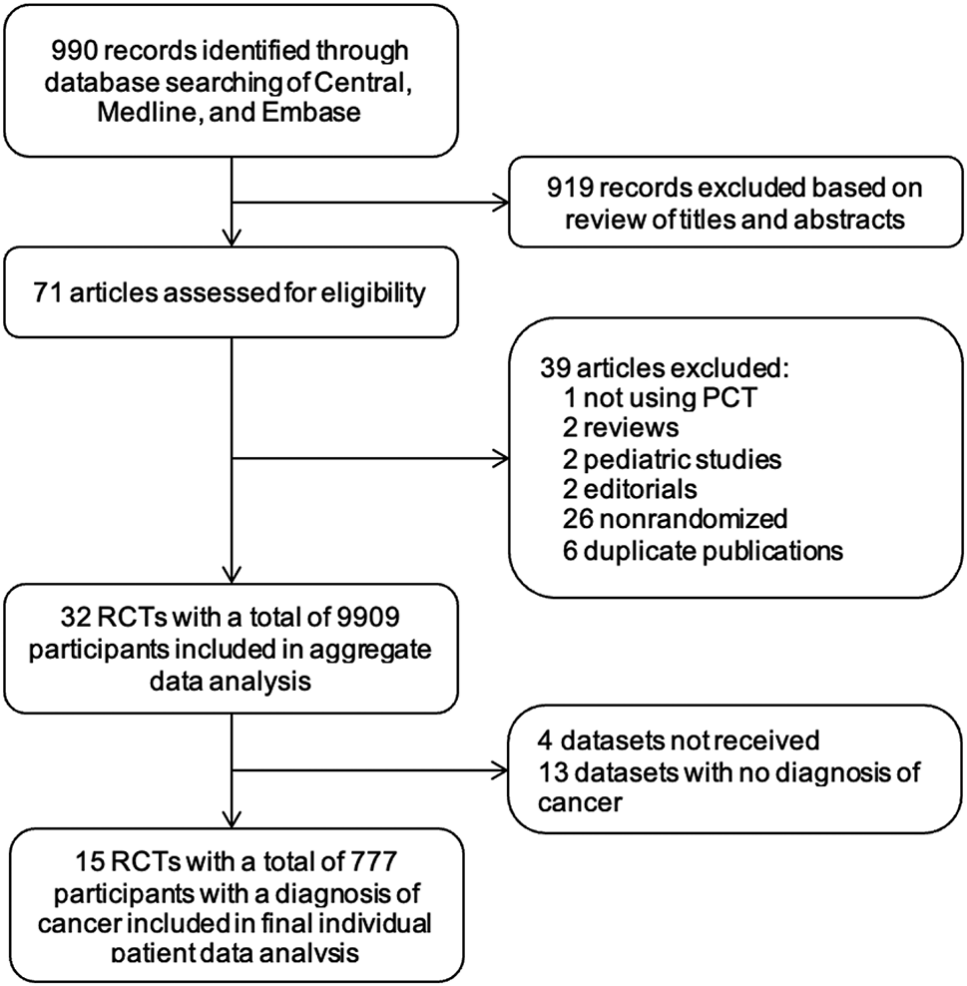
Study flow. Abbreviations: PCT, procalcitonin; RCT, randomized controlled trial

Through the systematic literature search, a total of 990 records were identified. Thereof, 32 randomized controlled trials were eligible for analysis. 13 trials were excluded due to the lack of information regarding cancer diagnosis, 4 datasets were not received and therefore did not provide sufficient clinical data. No additional trials meeting the inclusion criteria were found beyond 2017 with our search strategy. Hence, a total of 15 trials with 777 patients with a diagnosis of cancer were included in the final analysis (Fig. 1). Table 1 provides an overview of the 15 trials including country, clinical setting, infection diagnosis, type of PCT algorithm, number of patients and adherence to the PCT protocol. In brief, the analysis included trials that took place in eight different countries including Switzerland, France, Germany, Denmark, Belgium, Brazil, Australia and Italy. A total of eight trials were performed in the ICU and 6 trials were conducted in the ED or medical ward, while 1 trial took place in a primary care setting. Adherence to PCT-based algorithm varied between 46.3% and 97%.

### Baseline characteristics of included patients

Table 2 provides a summary of individual patients included in the trials stratified by randomization arm. There were 398 patients in the control group and 379 in the PCT group. The two arms were well balanced regarding baseline characteristics with no significant differences. Overall, more than half of the patients had a diagnosis of sepsis while the rest of patients had upper and lower respiratory infection including pneumonia.

### Primary efficacy and safety endpoints

Table 3 summarizes the effects of PCT-guided care on antibiotic exposure and mortality, as well as other endpoints.

**Fig. 2:**
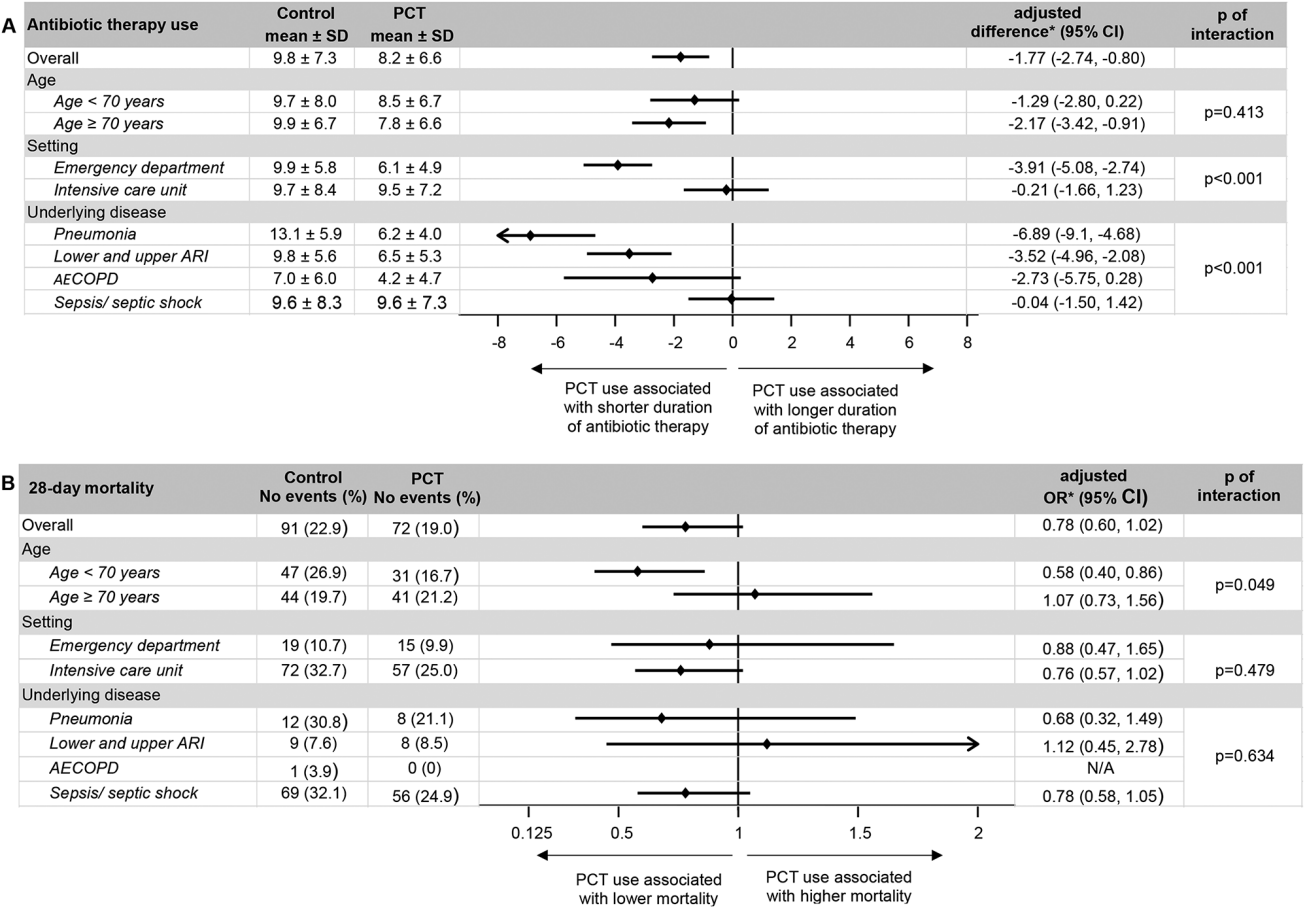
(**A**). Antibiotic therapy use defined as efficacy Endpoint and (**B**). 28-day mortality defined as safety endpoint. Abbreviations: AECOPD, acute exacerbation of chronic obstructive pulmonary disease; ARI, acute respiratory infection; CI, confidence interval; N/A, not applicable; OR, odds ratio; PCT, procalcitonin; SD, standard deviation; PCT, procalcitonin

Regarding efficacy, there was a 18% reduction in antibiotic exposure in patients randomized to PCT-guided care compared to usual care ([days] 8.2 ± 6.6 vs. 9.8 ± 7.3; adjusted difference, − 1.77 [95% CI, − 2.74 to − 0.80]; *p* < 0.001). A subgroup-analysis suggested no significant interactions by age (Fig. 2A). However, the effect of PCT use was more pronounced in patients in the emergency department (vs. intensive care, p for interaction < 0.001) and in patients with respiratory infections including pneumonia (vs. sepsis, p for interaction < 0.001).

Regarding safety, there were 72 deaths in 379 patients in the PCT-guided group (19.0%) compared to 91 deaths in 398 participants in the usual care group (22.9%) resulting in an adjusted OR of 0.78 (95% CI, 0.60 to 1.02). There was a significant effect in the subgroup analysis stratified by age with patients younger than 70 years randomized to the PCT group showing a significantly lower mortality compared to control group patients (adjusted OR 0.58 [95% CI, 0.40 to 0.86]; P for interaction = 0.049) (Fig. 2B).

Additionally, we also performed a sensitivity analysis excluding the largest trial by Bloos [33] and colleagues (Table S3). Overall, safety results remained similar, but the reduction in antibiotic treatment was much more pronounced (10.2 ± 6.9 vs. 7.0 ± 5.7 days, adjusted difference − 3.34 days (95%CI -4.47 to -2.21), *p* < 0.001).

### Additional secondary endpoints

Regarding need for ICU-care, there was no difference between both groups (adjusted difference, 1.08 [95% CI, 0.98 to 1.20]). Also, regarding length of hospital and ICU stay, there were no differences in between the PCT and the control group. These findings remained also consistent in the subgroup-analysis with no evidence for effect modification (Fig. 2 and Fig. S1).

## Discussion

This meta-analysis, based on individual data from 777 patients across 15 randomized trials worldwide, is, to our knowledge, the largest study examining the role of a PCT-guided protocol in directing antibiotic treatment for cancer patients. Results of this analysis suggest that among patients with cancer and suspected or proven respiratory infection or sepsis, the use of PCT to guide antibiotic decisions is both efficient, with reductions in antibiotic exposure, and safe, with possible positive mortality effects, particularly in younger patients. Several findings need further discussion.

PCT has been found to be a promising biomarker for the monitoring of patients with infections and an interesting candidate for guiding antibiotic stewardship. PCT concentrations increase in the blood stream after 6 h of bacterial infection. This increase is driven by various cytokines and is a response to pro-inflammatory mediators and proteins. The increase is most pronounced in patients with bacterial infections, and the absolute levels of PCT are correlated with disease severity and the risk of adverse clinical outcomes [34, 35]. Interestingly, the release of PCT is blocked by cytokines, typically released in response to viral infections including interferon-γ [36, 37]. For this reason, PCT has been found to be a more specific biomarker for bacterial infections while WBC and CRP are rather unspecific inflammatory markers [38–40]. In addition to the initial level, the kinetics of PCT over time also indicate the resolution of infection, which can be useful for monitoring purposes and for determining when to discontinue antibiotic treatment in conjunction with various clinical parameters. Importantly, decisions regarding antibiotic use in an individual patient are complex. Such decisions need to be based on the clinical presentation and pretest probability for severe infection in need of antibiotics, the overall risk of the patient and severity of infection and the biomarker level. Although the trials included in this analysis had different PCT protocols in place, the main concepts had many similarities and are also congruent with current PCT guideline recommendations [41–44]. In brief, most protocols defined bacterial infection to be unlikely and antibiotic treatment could be withheld or stopped in case of a low PCT level or a drop or PCT over time of > than 80–90% of the peak level [45]. Protocols use somewhat different cut-off thresholds regarding the clinical setting (i.e., for emergency department and medical ward patients a PCT cut-off of < 0.25 ug/L was used to recommend against antibiotic use while in intensive care PCT cut-off of < 0.5 ug/L was used to recommend discontinuation of treatment). Notably, the current analysis indicates that these PCT guidelines could also benefit cancer patients presenting with infections across different settings.

Several observational studies have previously looked at the association of PCT and risk of bacterial infection in patients with cancer and suspicion of infection [46–51]. In these studies, PCT had a high prognostic value and correlated with the risk of severe bacterial infection and adverse outcomes. However, only a few retrospective observational studies investigated the role of using PCT to guide antibiotics in patients with solid cancers. Dagher et al. [52] found a PCT cut-off < 0.25 µg/L to be associated with low likelihood of bacterial co-infection and shorter antibiotic course in patients with cancer with COVID-19, with similar mortality compared to control group patients. Liew et al. [53] found a significantly shorter antimicrobial stewardship program with carbapenem therapy when using PCT to support discontinuation or de-escalation in patients with cancers. Again, there was not increase in the risk for mortality. Our analysis aligns with these studies and suggests that the use of PCT leads to both improved antibiotic management and clinical outcomes.

For this analysis, we pooled individual data from patients with a diagnosis of cancer included in previous randomized PCT trials. These trials, however, differed in regard to the specific target population and patient diagnosis, the clinical setting, and the PCT protocols and guidelines used [54]. None of the trials had a specific focus on cancer, which is an important limitation of this work. Clearly, more interventional research in patients with cancer is needed to understand optimal use of PCT in this specific patient population.

Strengths of this meta-analysis include a comprehensive search strategy to identify relevant trials and acquire their individual patient data, resulting in a multicenter and multinational dataset with different settings. However, there are important limitations to this report. First, adherence to the PCT-guided algorithm was not perfect and ranged from 46 to 97% among the different trials. Low adherence may mask the effects of a PCT-guided algorithm [55]. Second, due to lack of other outcome data reported among trials, we were not able to investigate safety outcomes other than mortality, LOS [56] and the need for ICU-care. Further, although baseline characteristics of our patients were similar regarding age and gender our data did not differentiate between the specific type of cancer, since this information was not available. Also, we had no access to information regarding cancer treatment, which would have allowed to better characterize patients and perform further analyses. There is also risk of bias in the underlying trials and limited generalizability due to inclusion and exclusion criteria.

In conclusion, the result of this individual patient meta-analysis from 15 previous trials and 777 patients suggests that among patients with cancer and suspected or proven respiratory infection or sepsis, a PCT-guided antibiotic treatment decision results in reduced antibiotic exposure with a possible reduction in mortality, particularly among younger patients.

**Table 1:** Characteristics of included trials

First author (year)	Country	Setting, type of trial	Clinical diagnosis	Type of PCT algorithm and PCT cut- offs used (µg/L)	Patients included in trial	Patients with cancer diagnosis	Adherence to PCT protocol (%)
Annane [57]	France	ICU, Muticenter	Severe sepsis without overt source of infection and negative blood culture	Initiation and duration; R against AB: < 0.5 (< 0.25); R for AB: > 0.5 (> 5.0)	62	5	63
Bloss [33]	Germany	ICU, Multicenter	Severe sepsis or septic shock	Day 0, 1 = baseline; Discontinuation at day 4,7 and 10 if PCT value dropped at least 50%; other days R against AB: <1.0ng/ml or > 50% drop to previous value	1089	320	49.6
Briel [58]	Switzerland	Primary care, Multicenter	Upper and lower ARI	Initiation and duration; R against AB: < 0.25 (< 0.1); R for AB: > 0.25 (> 0.5)	458	1	85
Bouadma [59]	France	ICU, Multicenter	Suspected bacterial infections during ICU stay without prior AB (> 24 h)	Initiation and duration; R against AB: < 0.5 (< 0.25); R for AB: > 0.5 (> 1.0)	621	13	47
Christ-Crain [60]	Switzerland	ED, Single center	Lower ARI with X-ray confirmation	Initiation; R against AB: < 0.25 (< 0.1); R for AB: > 0.25 (> 0.5)	243	37	83
Christ-Crain [7]	Switzerland	ED, Medical ward, Single center	CAP with X-ray confirmation	Initiation and duration; R against AB: < 0.25 (< 0.1); R for AB: > 0.25 (> 0.5)	302	69	87
Kristoffersen [6]	Denmark	ED, Medical ward, Multicenter	Lower ARI without X-ray confirmation	Initiation and duration; R against AB: < 0.25; R for AB: > 0.25 (> 0.5)	210	7	59
Layios [61]	Belgium	ICU, Single center	Suspected infection	Initiation; R against AB: < 0.5 (< 0.25); R for AB: > 0.5 (> 1.0)	379	52	46.3
Nobre [62]	Switzerland	ICU, Single center	Suspected severe sepsis or septic shock	Duration; R against AB: < 0.5 (< 0.25) or > 80% drop; R for AB: > 0.5 (> 1.0)	79	10	81
Oliveira [63]	Brazil	ICU, Multicenter	Severe sepsis or septic shock	Discontinuation; Initial < 1.0: R againt AB: 0,1 at day 4; Initial > 1.0: R against: >90% drop	94	3	87.8
Schuetz [3]	Switzerland	ED, Medical ward, Multicenter	Lower ARI with X-ray confirmation	Initiation and duration; R against AB: < 0.25 (< 0.1); R for AB: > 0.25 (> 0.5)	1359	167	91
Shehabi [64]	Australia	ICU, Multicenter	Suspected Sepsis, undifferentiated infections	Duration; R against AB: < 0.25 (< 0.1) or > 90% drop	394	37	97
Stolz [10]	Switzerland	ED, Medical ward, single center	Exacerbated COPD	Initiation and duration; R against AB: < 0.25 (< 0.1); R for AB: > 0.25 (> 0.5)	208	41	Not reported
Stolz [8]	Switzerland, USA	ICU, Multicenter	VAP when intubated for > 48 h	Duration; R against AB: < 0.5 (< 0.25) or > 80% drop; R for AB: > 0.5 (> 1.0)	101	8	Not reported
Verduri [65]	Italy	ED, Medical ward, Multicenter	AECOPD	Initiation; R against AB:< 0.1; R for AB: > 0.5	178	7	95.5

Abbreviations: AB, antibiotic; ARI, acute respiratory infection; Bc, Blood culture; CAP, community-acquired pneumonia; COPD, chronic obstructive pulmonary disease; ED, emergency department; ICU, intensive care unit; RTI, respiratory tract infection; SIRS, systemic inflammation response system; VAP, ventilator-associated pneumonia

**Table 2:** Baseline characteristics stratified by PCT-group

Parameter	Control group (*n* = 398)	PCT group (*n* = 379)	*p*-value
Demographics			
Age [years] mean (SD)	69.8 (11.6)	69.1 (11.3)	0.38
Male gender, n (%)	257 (64.6)	253 (66.8)	0.52
Diagnosis, n (%)			
Pneumonia (CAP, VAP)	39 (9.8)	38 (10.0)	0.42
Lower and upper ARI	118 (29.6)	94 (24.8)	
AECOPD	26 (6.5)	22 (5.8)	
Sepsis/septic shock	215 (54.0)	225 (59.4)	
Laboratory assessment			
PCT day 0 [µg/L], mean (SD)	10.7 (29.9)	11.6 (49.4)	0.78
PCT cut-offs, n (%)			
PCT < 0.1 [µg/L]	37 (10.4)	46 (13.6)	0.14
PCT 0.1–0.25 [µg/L]	72 (20.3)	45 (13.3)	
PCT > 0.25–0.5 [µg/L]	35 (9.9)	38 (11.2)	
PCT > 0.5 -2.0 [µg/L]	57 (16.1)	56 (16.5)	
PCT > 2.0–10’000 [µg/L]	154 (43.4)	154 (45.4)	
CRP day 0 [µg/L], mean (SD)	157.5 (114.2)	187.4 (274.2)	0.06
Creatinine day 0 [µg/L], mean (SD)	127.7 (110.9)	142.3 (124.0)	0.12
Vital signs			
Temperature [°C], mean (SD)	37.8 (1.1)	37.8 (1.0)	0.28
Setting, n (%)			
Emergency Department	178 (44.7)	151 (39.8)	0.17
ICU	220 (55.3)	228 (60.2)	
Sepsis score			
SOFA score [points], mean (SD)	9.1 (3.8)	8.5 (3.9)	0.22
Additional support			
Vassopressor use, n (%)	173 (80.5)	191 (84.5)	0.26
Ventilation support, n (%)	157 (73.4)	167 (73.6)	0.96
Renal replacement, n (%)	52 (13.1)	52 (13.7)	0.79
Comorbidities, n (%)			
Liver failure	47 (12.5)	40 (11.2)	0.57
Congestive heart failure	90 (24.5)	88 (25.1)	0.85
Central nervous system	34 (9.6)	29 (8.5)	0.62
End-stage renal disease	96 (26.2)	77 (21.9)	0.18
Peripheral artery disease	27 (8.2)	31 (10.0)	0.42
Hypertension	55 (70)	46 (62)	0.33
Diabetes mellitus	76 (20.8)	74 (21.1)	0.9
Immunosupression	8 (4.6)	8 (4.3)	0.86

*Abbreviations*: CAP, community acquired pneumonia; VAP, ventilator associated pneumonia; ARI, acute respiratory infection; AECOPD, acute exacerbation of chronic obstructive pulmonary disease; CRP, c-reactive protein; PCT, procalcitonin; SD, standard deviation; SOFA, sepsis-related Organ Failure Assessment; ICU, intensive care unit

**Table 3:** Clinical endpoints

Outcomes	Control group (*n* = 398)	PCT group (*n* = 379)	Adjusted OR or difference (95% CI)*, *p*-value
28- days mortality, n (%)	91 (22.9)	72 (19.0)	0.78 (0.60, 1.02), *p* = 0.069
Need for ICU care, n (%)	236 (59.3)	241 (63.6)	1.08 (0.98, 1.20), *p* = 0.125
Antibiotic therapy [days], mean (SD)	9.8 (7.3)	8.2 (6.6)	**-1.77 (-2.74**,** -0.80)**,***p* < 0.001**
Length of hospital stay [days], mean (SD)	20.9 (18.1)	24.2 (22.5)	1.85 (-0.88, 4.57), *p* = 0.184

Abbreviations: CI, confidence interval; ICU, intensive care unit; OR, odds ratio; PCT, procalcitonin; SD, standard deviation

*Multivariable hierarchical regression with outcomes of interest as dependent and trail as a random effect

Statistically significant results are diplayed in bold

## Electronic supplementary material

Below is the link to the electronic supplementary material.

Supplementary Material 1

Supplementary Material 2

## Data Availability

The datasets analyzed during the current study are available from the corresponding author on reasonable request.
