# Supplementary material for: Procalcitonin-guided antibiotic treatment in patients with cancer: a patient-level meta-analysis from randomized controlled trials
Source: BMC Cancer. 2024 Nov 28;24:1467. doi: 10.1186/s12885-024-13160-2 (PMC11606202; doi:10.1186/s12885-024-13160-2)
Supplement: Supplementary file 2 — Supplementary Material 2 [file 12885_2024_13160_MOESM2_ESM.pdf]

Supplementary Material

Article Procalcitonin-guided antibiotic treatment in patients with cancer: a patient-level meta-analysis from randomized controlled trials

Claudia Gregoriano*[1], Yannick Wirz*[1], Ashley Heinsalo*[1], Djilali Annane[3], Konrad Reinhart[4], Lila Bouadma[6], Mirjam Christ-Crain[7], Kristina B. Kristoffersen[8], Pierre Damas[9], Vandack Nobre[10], Carolina F. Oliveira[10], Yahya Shehabi[11], Daiana Stolz[12], Alessia Verduri[13], Beat Mueller[1,2], and Philipp Schuetz[1,2]

*Equally first
[1] Medical University Department, Kantonsspital Aarau, Aarau, Switzerland
[2] Department of Clinical Research (DKF) and Faculty of Medicine, University of Basel, Switzerland
[3] IHU PROMETHEUS, Raymond Poincaré Hospital (APHP), INSERM, Université Paris Saclay Campus Versailles, Paris, France.
[4] Department of Anesthesiology and Intensive Care Medicine, Jena University Hospital, Jena, Germany.
[6] Médecine intensive-réanimation, AP-HP, Hôpital Bichat-Claude Bernard, Paris, France.
[7] Division of Endocrinology, Diabetology and Clinical Nutrition, University Hospital Basel, Basel, Switzerland
[8] Department of Oncology, Aarhus University Hospital, Aarhus, Denmark.
[9] Department of Intensive Care, University Hospital Liège, Liège, Belgium.
[10] Department of Internal Medicine, Medical School and University Hospital, Universidade Federal de Minas Gerais, Belo Horizonte, Brazil.
[11] Department of Intensive Care, Monash Medical Centre, Melbourne, VIC, Australia.
[12] Clinic of Respiratory Medicine and Faculty of Medicine, University of Freiburg, Freiburg, Germany
[13] Respiratory Unit, Department of Surgical and Medical Sciences, University of Modena and Reggio Emilia, Modena, Italy.

Table S1. Definition of ARI subtype

**ARI subtype**	**Definition**
Upper respiratory infection	Clinical diagnosis of common cold, rhinosinusitis, pharyngitis, tonsillitis, otitis media or other unspecific upper respiratory infection; no additional diagnostic tests required. Definitions were similar among the two primary care trials.
Lower respiratory tract infection	Presence of at least one respiratory symptom (cough, sputum production, dyspnea, tachypnea, pleuritic pain) plus at least one finding during auscultation (rales, crepitation), or one sign of infection (core body temperature >38.0°C, shivering, leukocyte count >10`000cells/uL or <4`000cells/uL) independent of antibiotic pre-treatment
Acute bronchitis	Lower respiratory tract infection without infiltrate in the absence of an underlying lung disease or focal chest signs and infiltrates on chest X-ray
Community-acquired pneumonia (CAP)	Lower respiratory tract infection with a new infiltrate in the Chest X-ray admitted from the community. In the Kristoffersen trial, chest X-ray signs of pneumonia were not required for inclusion in the study.
Hospital-acquired pneumonia	Lower respiratory tract infection with a new infiltrate in the Chest X-ray in a patient in a hospital setting for at least 48–72hours
Ventilator-associated pneumonia (VAP)	ICU patients intubated for mechanical ventilation for >48 h with all of the following criteria: 1) clinically diagnosed with a new or persistent infiltrate on chest radiography associated with at least two of the following: purulent tracheal secretions, temperature >38C or, leukocyte count >11,000 mL or ,3,000 mL
Exacerbation of asthma	Episodic symptoms of airflow obstruction, which are at least partly reversible, as assessed by lung-function tests
Exacerbation of chronic obstructive pulmonary disease (ECOPD)	Sustained worsening of the patient’s condition, from the stable state and beyond normal day-to-day variations, that is acute in onset and necessitates a change in regular medication in a patient with underlying COPD; defined by post-bronchodilator spirometric criteria according to the GOLD-guidelines; in patients with a clinical history of COPD and smoking, lung function testing at the time of inclusion was not mandatory

Legend: ARI, acute respiratory infection; COPD, chronic obstructive pulmonary disease;

Figure S1. Forest-plots of further secondary outcomes

Need for ICU care	Control No events (%)	PCT No events (%)	adjusted difference* (95% CI)	p of interaction
Overall	236 (40.7)	241 (63.6)	1.08 (0.98, 1.20)	
Age				
Age < 70 years	124 (70.9)	137 (73.7)	1.06 (0.95, 1.18)	p=0.982
Age ≥ 70 years	112 (50.2)	104 (53.9)	1.08 (0.91, 1.30)	
Setting				
Emergency department	21 (11.8)	16 (10.6)	0.85 (0.46, 1.58)	N/A
Intensive care unit	215 (100)	225 (100)	N/A	
Underlying disease				
Pneumonia	8 (20.5)	4 (10.5)	0.51 (0.17, 1.56)	p=0.316
Lower and upper ARI	9 (7.6)	10 (10.6)	1.40 (0.59, 3.29)	
acute exacerbation of COPD	4 (15.4)	2 (9.1)	0.59 (0.12, 2.93)	
Sepsis/ septic shock	215 (100)	225 (100)	N/A	

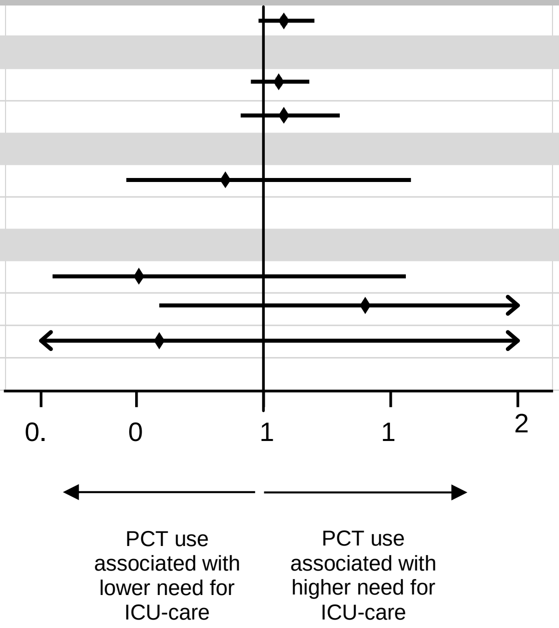

Length of hospital stay	Control mean ± SD	PCT mean ± SD	adjusted difference* (95% CI)	p of interaction
Overall	20.9 ± 18.1	24.2 ± 22.5	1.85 (-0.88, 4.57)	
Age				
Age < 70 years	22.9 ± 18.9	25.1 ± 23.1	0.64 (-3.75, 5.03)	p=0.402
Age ≥ 70 years	19.6 ± 17.4	23.3 ± 21.9	2.87 (-0.61, 6.35)	
Setting				
Emergency department	12.8 ± 10.0	13.1 ± 9.1	-0.19 (-2.20, 1.82)	p=0.273
Intensive care unit	29.2 ± 20.6	32.7 ± 25.8	3.06 (-1.80, 7.92)	
Underlying disease				
Pneumonia	18.7 ± 10.6	15.0 ± 8.6	-3.69 (-7.94, 0.55)	p=0-126
Lower and upper ARI	11.4 ± 9.8	12.1 ± 9.7	0.07 (-2.43, 2.56)	
acute exacerbation of COPD	13.0 ± 9.5	15.6 ± 7.4	2.59 (-2.56, 7.73)	
Sepsis/ septic shock	29.3 ± 20.7	32.9 ± 25.9	3.46 (-1.45, 8.37)	

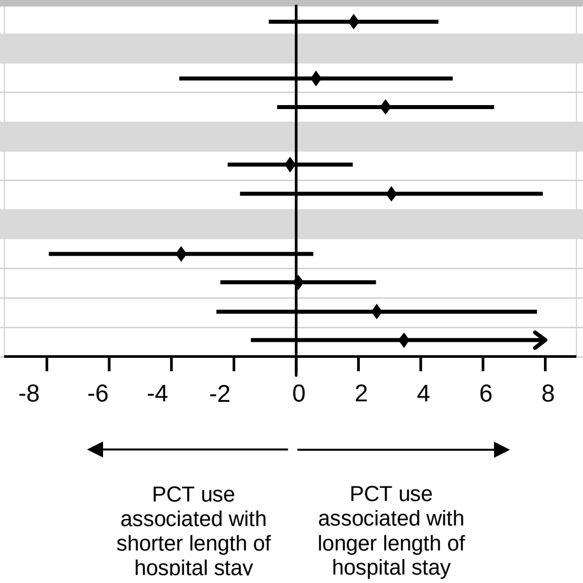

Table S2. Further characteristics of included trials

First author (year)	allocation concealment	blinded outcome assessment	follow-up for mortality	Adherence to PCT algorithm in PCT group	Follow up
Annane (2013)	yes (central randomization)	no	58/58 (100%)	63% adherence	LOS
Bouadma (2010)	yes (central randomization)	yes	393/394 (100%)	47% adherence	28 days and 60 days
Bloss (2016)	yes (central randomization)	no	1076/1089 (98.8%)	49.6% adherence	28 days and 90days
Briel (2008)	yes (central randomization)	yes	454/458 (99%)	454/458 (99%)	28 days
Christ-Crain (2004)	no (alternating weeks)	no	230/243 (95%)	83% adherence	10 -14 (-28)d follow up
Christ-Crain (2006)	yes (sequentially numbered, opaque, sealed envelopes)	no	300/302 (99%)	87% adherence	56 d follow up
Kristoffersen (2009)	yes (central randomization)	no	210/210 (100% until discharge)	59% adherence	LOS
Layios (2012)	not reported	yes	509/509 (100%)	46.3% adherence	ICU LOS
Nobre (2008)	yes (sequentially numbered, opaque, sealed envelopes)	no	52/52 (100%)	81% adherence	28d and LOS
Oliveira (2013)	yes (central randomization)	no	94/94 (100%)	87.8% adherence	28 days
Schuetz (2009)	yes (central randomization)	yes	1358/1359 (100%)	91% adherence	28 days
Shehabi (2014)	yes (central randomization)	yes	394/394 (100%)	97% adherence	LOS and 90 days
Stolz (2007)	yes (sequentially numbered, opaque, sealed envelopes)	yes	208/208 (100%)	not reported	14 days - 6 month
Stolz (2009)	yes (sequentially numbered, opaque, sealed envelopes)	no	101/101 (100%)	not reported	28 days
Verduri (2015)	yes (central randomization)	no	178/178 (100%)	95.5% adherence	6 Months

Abbreviations: LOS, Length of overall stay

Table S3: Sensitivity analysis of clinical endpoints (without Bloos trial)

Outcomes	Control group (n=244)	PCT group (n=213)	Adjusted OR or difference (95% CI)*, p-value
**28- days mortality, n (%)**	38 (15.6)	30 (14.1)	0.85 (0.56, 1.30), p=0.453
**Need for ICU care, n (%)**	82 (33.6)	75 (35.2)	093 (0.76, 1.13), p=0.448
**Antibiotic therapy [days], mean (SD)**	10.2 (6.9)	7.0 (5.7)	**-3.34 (-4.47, -2.21), p<0.001**
**Length of hospital stay [days], mean (SD)**	15.2 (13.5)	14.4 (11.6)	-1.40 (-3.72, 0.93), p=0.238

Abbreviations: CI, confidence interval; ICU, intensive care unit; OR, odds ratio; PCT, procalcitonin; SD, standard deviation

*Multivariable hierarchical regression with outcomes of interest as dependent and trail as a random effect.
